# Forensic analysis of deaths from train and subway surfing

**DOI:** 10.1007/s00414-025-03551-w

**Published:** 2025-06-20

**Authors:** Alberto Amadasi, Larissa Amadasi, Luca Berti, Lars Oesterhelweg

**Affiliations:** 1 Institute of Legal Medicine and Forensic Sciences, Charité - Universitätsmedizin Berlin, Turmstrasse 21, 10559 Berlin, Germany; 2 Department of Medical and Surgical Sciences, University of Bologna, Via Irnerio 49, Bologna, 40126 Italy

**Keywords:** Train surfing, Electrocution, Forensic pathology, Traumatic injuries, Fatal accidents, Social media influence

## Abstract

Train surfing, the practice of riding on the exterior of moving trains or subways, is an increasingly prevalent phenomenon, particularly among urban youth, and is partly fueled by social media exposure. While it provides an adrenaline rush, it presents significant risks, including electrocution, falls, and collisions with objects. This study aims to investigate the forensic aspects of fatalities associated with train surfing incidents, focusing on cases examined by the Institute of Forensic Medicine at Charité University in Berlin between 2010 and 2024. A total of 15 fatalities resulting from train surfing were analyzed, all involving male victims aged 15–24, with the majority of incidents occurring during the summer months. The primary cause of death was traumatic injury (11 cases), resulting from falls or impacts with obstacles, while electrocution (particularly from high-voltage contact or electric arcs) was responsible for the remaining 4 fatalities. In many cases, victims sustained multiple injuries, including isolated cranial trauma and polytrauma involving the head. Forensic findings, such as electrical burn marks and blunt force injuries, were crucial in differentiating between electrocution and trauma. Toxicological analyses revealed the presence of substances like alcohol, THC, and cocaine in several cases, underscoring the role of substance use in these incidents and in the reconstruction of events. This research emphasizes the need for comprehensive forensic protocols to address such cases, as train surfing remains a significant public safety concern.

## Introduction

Train or subway surfing, the act of riding on the exterior of moving trains or subway cars, is a dangerous and often illegal activity that has garnered significant attention in recent years. While the practice has existed for several decades, it has evolved substantially, partly due to the influence of social media platforms where individuals post videos of their perilous stunts. What was once a subcultural activity has increasingly become a widespread phenomenon, particularly among urban youth seeking to experience extreme thrills [1–4]. This growing trend has raised considerable concern among transportation authorities, law enforcement, and safety experts, as the risks associated with train surfing are not only life-threatening but also disruptive to public transportation systems. In Germany, train surfing is considered an administrative offense under § 28 para. 1 no. 9 of the General Railway Act (AEG) and may result in fines of up to 50,000 euros. Offenders can also face up to ten years of imprisonment under § 315 of the Criminal Code (StGB) for endangering rail, maritime, and air traffic [5].

The motivations behind train surfing are multifaceted, shaped by a complex interplay of social, psychological, and cultural factors. For many participants, the primary appeal lies in the intense adrenaline rush that accompanies the activity. The challenge of riding on the exterior of a moving train—often at high speeds—offers an irresistible thrill, especially for young people eager to test their limits. Additionally, the rise of social media platforms like Instagram, TikTok, and YouTube has fueled the desire for fame and validation, with social networks taking action by deleting thousands of videos documenting these dangerous stunts [6].

Despite the allure, the dangers of train surfing are severe and often fatal. The risks associated with riding on the exterior of a moving train include the potential for falls from great heights, collisions with overhanging objects, or electrocution from high-voltage power lines. In many cases, failed stunts lead to catastrophic consequences, resulting in serious injuries or death [7–9]. Furthermore, train surfers are at risk of being struck by other trains or vehicles or becoming entangled in the train’s mechanical components. The high-speed nature of the activity further amplifies the potential for harm, with even a brief lapse in attention or misstep leading to life-threatening outcomes.

The increasing frequency of these incidents, alongside the global spread of the trend, has raised alarms among authorities. Public transportation systems now face the challenge of curbing this dangerous behavior while ensuring the safety of passengers and the integrity of the trains.

In both clinical and forensic settings, numerous cases of injuries have been reported, the majority of which involve electrical burns and polytrauma resulting from accidents related to train surfing [10]. The aim of this forensic case series is to provide a comprehensive overview of the differential diagnosis and medico-legal aspects encountered in fatalities related to railway accidents. This study analyzes various cases of deaths resulting from train surfing, examining the causes, incident dynamics, and forensic diagnoses in these tragic situations.

## Materials and methods

For the execution of this study, judicial autopsy cases conducted at the Institute of Forensic Medicine at Charité University in Berlin were examined over the period from 2010 to 2024. The first selection criterion was unnatural death occurring within the context of rail or subway-related incidents. A total of 398 cases were identified, from which deaths occurring in the context of train/subway surfing and/or climbing were specifically selected. This study only included victims confirmed to have been active train surfers, as determined through police investigations. In this context, “active” refers to the voluntary and independent act of climbing onto the exterior of a moving train to engage in surfing. Falls from moving trains unrelated to surfing were not considered valid for inclusion in this study; evidence had to clearly demonstrate that train surfing was the primary cause of the incident.

In total, 15 cases were identified. For each case, data including age, sex, type of death, time of death, time of incident, cause and manner of death, and the results of toxicological investigations were recorded. Postmortem radiological investigations, including 3D reconstructions, were also analyzed for each case. In instances of traumatic injury, severity was rated according to the AIS (Abbreviated Injury Scale) [11] Trauma Score.

The aim of this study is to provide insights for potential differential diagnosis in a relatively uncommon context, such as subway/train surfing within railway-related accidents. Given the significant role of global social media in recent times, these may be cases that forensic pathology is increasingly likely to encounter.

## Results

The victims ranged in age from 15 to 24 years, with an average age of 17.2 years (excluding one 24-year-old, all other victims were between 15 and 19 years old). All victims were male. In 13% of the cases, a survival period was observed after the incident, lasting up to approximately 35 h. The majority of incidents, 86% (13 out of 15), occurred between June and October, with one case in March and another in April. Toxicological investigations revealed that 40% of the cases were negative for alcohol and/or drug consumption. In 46% of the cases (7 out of 14), blood alcohol concentrations were found to range between 0.3 and 2.3‰. Additionally, it was found that at the time of the accident, the victims were under the influence of THC (detected in 3 cases) and cocaine (detected in 2 cases).

A summary of the causes of death, the mechanisms of the accident, and the main characteristics of the injuries is provided in Table 1.

**Table 1:** Summary of the 15 cases

Cause of death	Number of cases	Mechanism of the accident	Main Characteristics of the injuries
Electrocution (contact)	2	Direct contact with high-voltage components	Electric mark on the hand and foot
Electrocution (electric arcing)	1	High-voltage electric field without direct contact	Second- and third-degree burns on approximately 80% of the body
Cranio-cerebral trauma and electrocution (electric arcing)	1	Impact against an obstacle and high-voltage electric field	Cranial and cerebral injuries and carbonization of the body
Cranio-cerebral trauma	2	Impact against an obstacle	Multiple skull fractures and brain injuries
Cranio-cerebral trauma	1	Fall without direct impact with the train.	Multiple skull fractures and brain injuries
Cranio-cerebral and neck trauma	1	Fall without direct impact with the train.	Multiple skull fractures, brain injuries and cervical spine fractures
Mulitple injuries without involvement of the head	1	Fall with impact with the train and being run over	Lower limb amputations, abdominal injuries
Multiple injuries including cranioencephalic injuries	6	Fall with impact with the train and being run over	Multiple injuries (reported in Fig. 4)

**Fig. 1:**
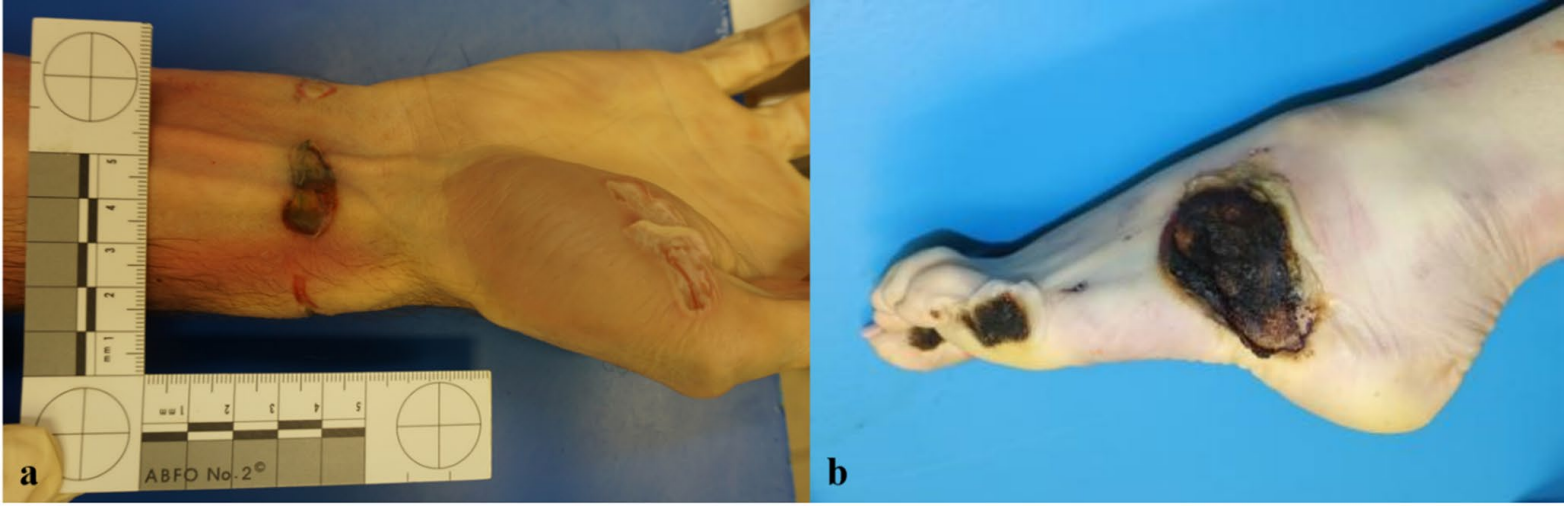
Electrical contact marks on the right hand and wrist (**a**), with marks from electrical current exit on the left foot (**b**)

In two cases, the cause of death was attributed to the victim’s direct contact with electrical components near the high-voltage train, resulting in involuntary direct contact (or at least an attempt to prevent loss of balance) and subsequent death by electrocution due to the transmission of electrical current through the body. In both cases, the autopsy revealed distinct electrical burn marks on the hand/wrist and foot (Fig. 1). The current marks on the skin exhibited extensive epidermal necrosis, intraepidermal blistering, and elongation of epithelial cell nuclei, with signs of focal metallization. Acute necrosis affected both the epidermis and dermis, accompanied by intense nuclear elongation and blister formation. Additionally, there was a sparse infiltration of leukocytes in the deeper dermis and adjacent subcutaneous tissue.

In one case, the victim survived for approximately 3 h after the incident, having suffered a cardiac arrest at the scene, been temporarily resuscitated successfully, and then transported to a hospital.

In another case, death was caused by the transmission of high-voltage electric current, leading to the creation of an electric arc, which resulted in second- and third-degree burns covering approximately 75% of the body.

**Fig. 2:**
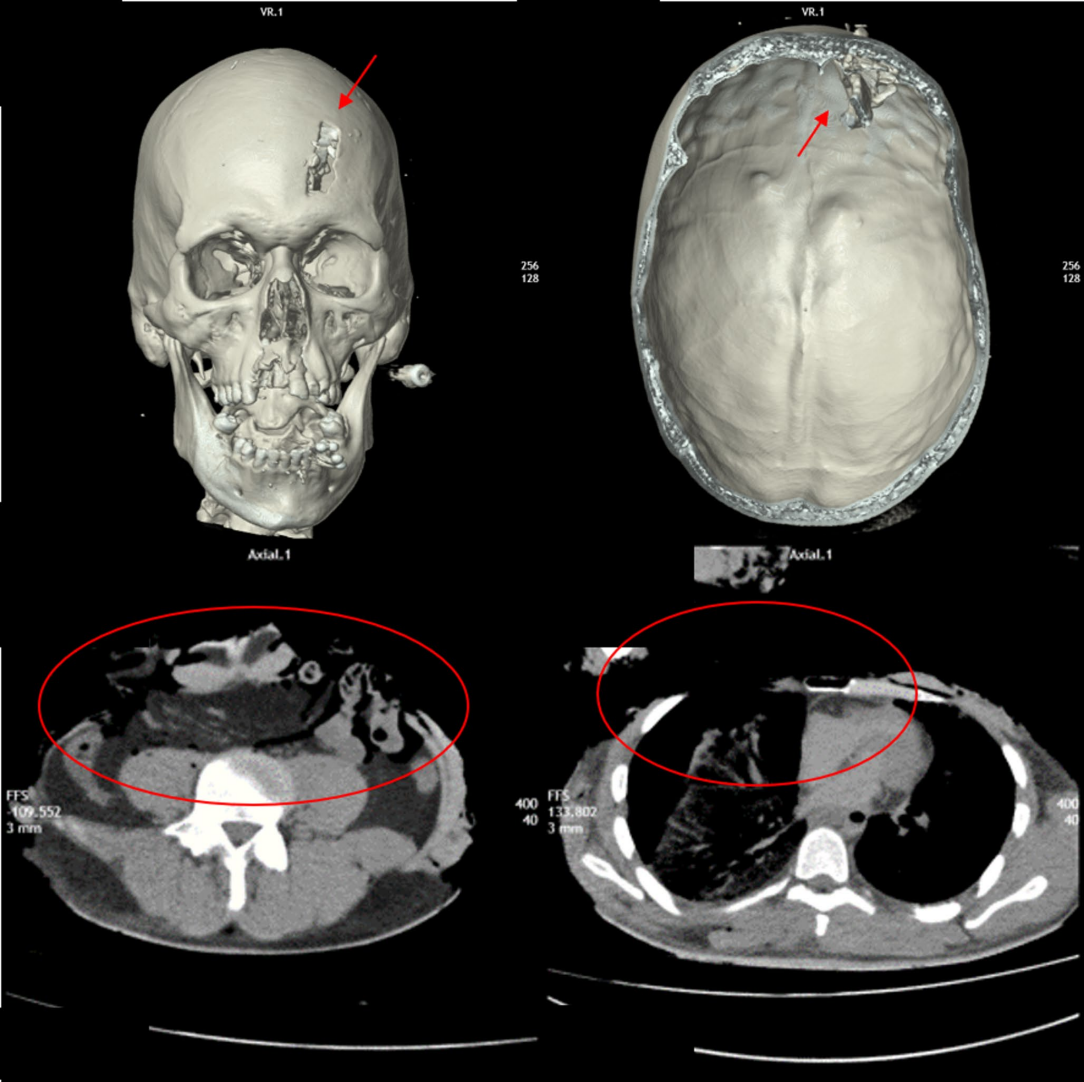
Clockwise: Depressed skull injury from impact against a protruding portion of an obstacle (red arrows), followed by the creation of electric arc burns and extensive subsequent carbonization of the body, with large areas of continuous injury on the right anterior thoracic and bilateral abdominal regions (red circles)

In one case, a combined injury was observed: cranio-encephalic trauma due to impact with an obstacle (likely the protruding part of a sign), resulting in a depressed, imprinted skull fracture of the frontal bone. This was followed by the creation of a high-voltage electric arc and subsequent widespread charring of the body (Fig. 2).

**Fig. 3:**
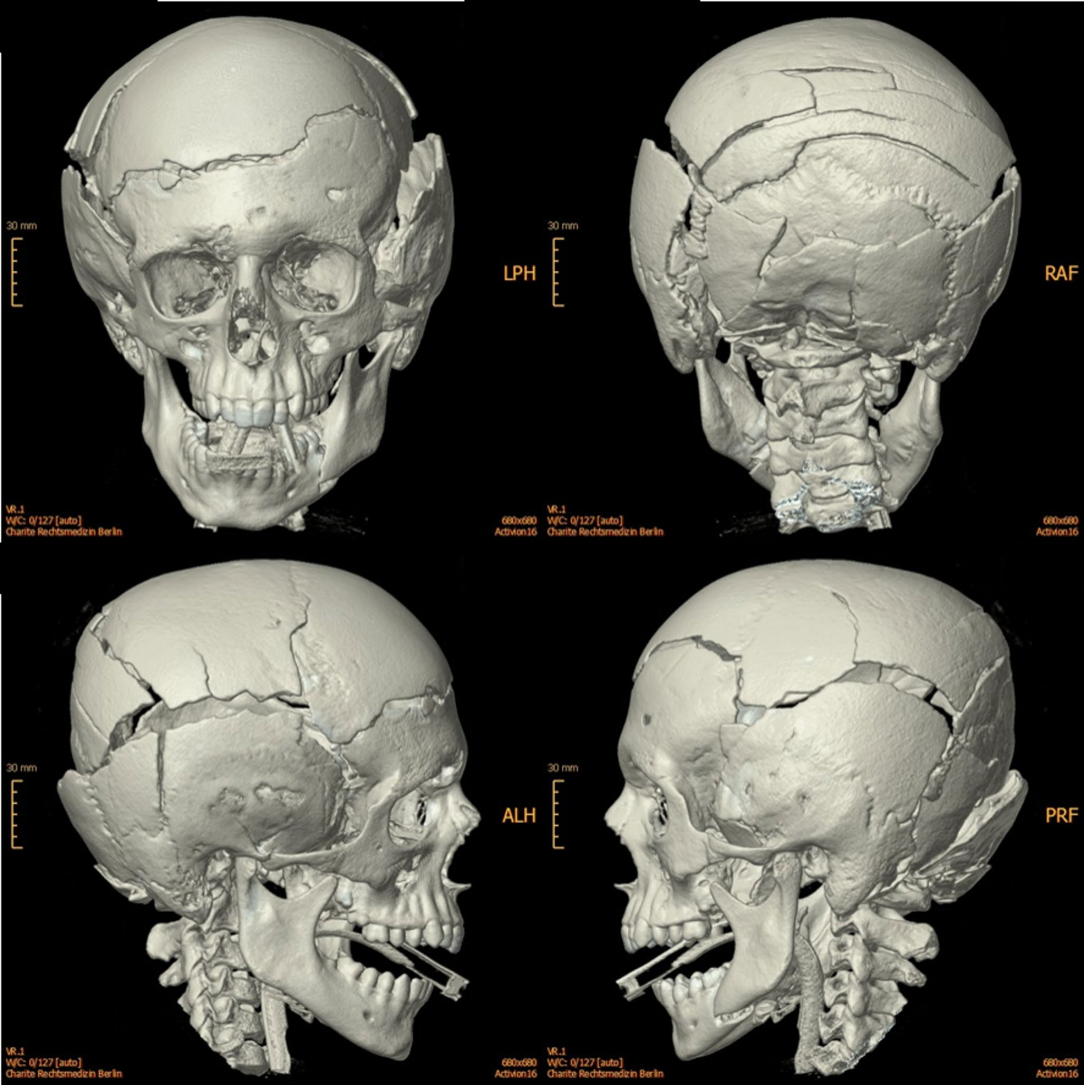
Multiple cranial fractures from impact against an obstacle (bridge)

In the remaining 11 cases (73%), death was caused by impact injuries. In two of these cases, death resulted from skull-brain injuries caused by impact with an obstacle: a signpost and a bridge (Fig. 3). In one case, the complex and extensive cranial fracture pattern exhibited the effects of a high-speed impact against a large, rigid surface at the occipital cranial region.

**Fig. 4:**
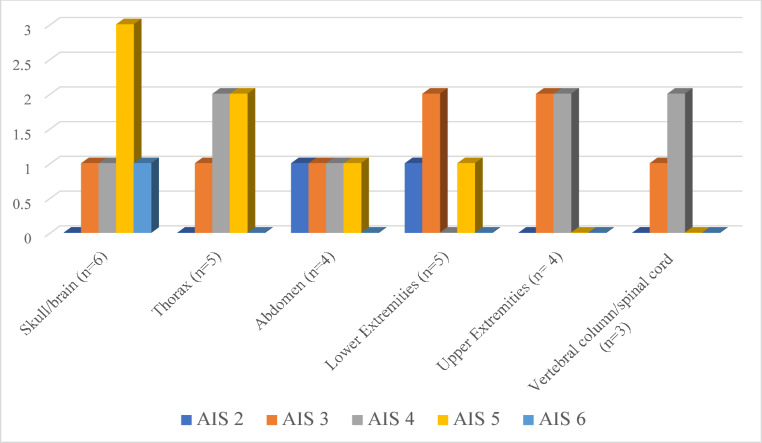
AIS severity score of the in cases of death due to multiple lesions with head involvement (*n* = 6)

In two other cases, the cause of death was attributed to cranioencephalic injuries (one of which was associated with a cervical vertebral fracture and spinal cord transection), resulting from falling off the train without direct contact with the train itself. Only in one case was there no involvement of the skull; the victim fell into a gap between two carriages and was subsequently run over by the train, leading to partial bilateral amputation of the lower limbs and abdominal injuries. In the remaining 6 cases (40%), the cause of death was due to polytrauma affecting multiple body areas (Fig. 4).

In these cases, the severity of the injuries was assessed using the AIS (Abbreviated Injury Scale) [11] Trauma Score, which indicated that in most cases, the injuries were indeed of a serious nature, particularly regarding the cranioencephalic region. The injury patterns varied depending on the case, but they were all intrinsically linked to the typical consequences of high-speed impacts with heavy mass vehicles (such as trains), as well as the potential for the body to be run over by the train and its mechanical components, leading to particularly severe or even catastrophic injuries.

## Discussion

The phenomenon of train surfing, particularly on subways or trains, is an increasingly concerning issue, especially given the rise in exposure through social media. The risks associated with this activity are substantial, often leading to fatal outcomes that require precise forensic analysis for both the cause of death and differential diagnosis. The study confirmed that train surfing predominantly affects young individuals (with the majority being minors) and is more common among males [8–10]; in this collection of cases, all victims were male. This series of cases highlights the typical aspects that forensic pathology must address and underscores the need for focused in-depth analysis.

### Causes of death in train/subway surfing accidents: not a simple “railway accident”

The first issue concerns the cause of death. A potential cause of death in train surfing incidents is electrocution due to exposure to the high-voltage electrical systems used to power trains and subways. These systems typically operate with direct current (DC) or alternating current (AC) at voltages that can cause immediate and fatal injury. Trains and subways are powered by overhead contact lines (called “catenaries” or “trolleys”) that carry high-voltage electricity (typically between 600 and 3,000 volts in railway systems). If a person comes into direct contact with these power lines, they may experience a fatal electric shock. Moreover, individuals close to high-voltage power lines may be affected by “contact voltage” or “step voltage.” Contact voltage occurs when a person touches an electrically charged object, while step voltage occurs when a person walks on a surface where electrical current has created a potential difference [7–9]. The electricity quickly flows through the body, causing severe tissue damage and internal organ failure. Death may also occur from an electric arc or exposure to the electric field. Even if a person does not make direct contact with the power cables, they could be struck by an electric arc, which occurs when electricity jumps through the air from the person to the power line [12–16].

The danger extends to those who are not in direct contact with the power source but are within proximity to these high-voltage systems. Electric arcs and step voltage can cause fatal injuries, even without direct contact, by discharging electricity through the air or the ground. The study found that in cases of electrocution, victims were electrocuted either by directly touching high-voltage cables or by being struck by electric arcs. Forensic evidence, particularly electrical burn marks, was crucial in determining the cause of death. In one case, second- and third-degree burns over a large portion of the body were found, consistent with electric arc injuries. The types of injuries provide medico-legal information regarding the involvement of electrical injury and the mechanism involved.

In addition to electrical risks, there is also the danger of falling or being struck by other objects or trains, which could cause further injuries or death [17, 18]. These injuries can be particularly destructive (typical of those related to railway or aviation accidents [19]), to the point of making identification difficult [20]. The most frequent cause of death in the analyzed cases resembles what has been observed in cases of railway accidents involving pedestrians, as a result of trauma from falls or impact injuries, either from falling from the moving train or being struck by obstacles such as signposts or bridges. In some cases, multiple injuries, such as cranio-encephalic trauma (head injuries) and polytrauma, resulting from various impacts, were the primary contributors to the victims’ deaths. Trauma typically involved the head and spine, including fractures of the cervical vertebrae and spinal cord transection. This injury pattern is consistent with those found in railway accidents, whether accidental or suicidal in nature [17, 18, 21, 22].

### Dynamics of fatal incidents and impact on diagnosis

The dynamics of these accidents can be complex, often involving a combination of electrical burns, traumatic impact, and mechanical injuries. In many cases, individuals lose their balance or fail to maintain grip on the moving train, increasing the risk of falls and trauma. The unpredictable movement of the train (due to acceleration, braking, and sharp turns) further exacerbates the likelihood of such incidents.

Forensic investigation of train surfing fatalities requires careful attention to injury patterns and the differentiation between electrocution and blunt trauma injuries. Electric burns and internal injuries caused by electrocution typically show distinctive forensic markers, including electrical burn marks and signs of internal organ failure. In contrast, impact injuries are characterized by extensive blunt force trauma. The analysis of injury severity, as often encountered in cases of pedestrian collisions with trains, reveals that even in these cases, injuries are often very severe or destructive, frequently resulting in death at the scene. Injury analysis, however, is critical for understanding the dynamics of the event. For example, cases of cranial injury resulting from impact against a large, rigid surface (e.g., a bridge) and the resulting cranio-cerebral injury pattern, as well as depressed cranial fractures typical of impacts against relatively small surfaces, are often associated with electrical injuries such as electrical burn marks or carbonization of the body. It must be emphasized that traumatic injuries, particularly cranio-encephalic injuries, can result from various dynamics: direct impact against an external rigid surface, falling to the ground, the effects of railway traffic, or a combination of these. A proper analysis of the injuries can provide essential elements for the accurate reconstruction of events. For example, combined skull-brain and neck injuries, as well as fractures of the upper and lower limbs, may overlap with those observed in typical falls from heights [23, 24].

In this context, radiological investigation also plays a crucial role, with the possibility of 3D reconstruction of cranial injuries, an essential element for reconstructing the cranio-cerebral injury dynamics [25]. Forensic pathologists must also consider the presence of other factors, such as substance use, as indicated by toxicological reports. This study revealed that 40% of victims tested negative for alcohol or drugs, while others had moderate alcohol levels or traces of substances like THC and cocaine. The involvement of drugs or alcohol can influence the victim’s ability to navigate the roof of the train and the severity of the trauma sustained, complicating the diagnosis and reconstruction of events.

### Differential diagnosis in forensic investigations

Differentiating the cause of death in train surfing incidents is critical in establishing the manner of death. A differential diagnosis should carefully assess:

- Electrocution markers: Presence of electric burns and organ damage typical of high-voltage electrocution.
- Impact trauma: Identifying blunt force trauma, fractures, or internal injuries consistent with falls or strikes.
- Polytrauma: Evaluating cases with multiple injuries across the body, where trauma from various sources (e.g., falling, getting run over by the train) may contribute to death.
- Environmental factors: Forensic pathologists must also consider whether the victim was exposed to electric arcs or step voltage, even without direct contact with high-voltage components.

The diagnostic challenge in these cases is magnified by the rapid onset of death and the often severe destruction of tissue, requiring careful post-mortem examination and toxicology reports.

## Conclusion

Train and subway surfing is an extremely hazardous activity that often leads to fatal outcomes, primarily resulting from electrocution or traumatic injuries. Forensic pathologists must carefully evaluate a variety of factors when investigating these deaths, including the presence of electrical injuries, blunt trauma, and the potential for polytrauma. Properly distinguishing between electrocution and trauma is crucial for determining the exact cause of death and making accurate legal conclusions. As the popularity of train surfing increases, particularly among younger individuals, it is expected that forensic pathologists will encounter more such cases in the future. This trend highlights the urgent need for the development of standardized protocols for investigating these incidents to ensure consistency and precision in forensic analyses.

## Data Availability

The Authors confirm that the data supporting the findings of this study are available within the article.
